# Safety, activity, and molecular heterogeneity following neoadjuvant non-pegylated liposomal doxorubicin, paclitaxel, trastuzumab, and pertuzumab in HER2-positive breast cancer (Opti-HER HEART): an open-label, single-group, multicenter, phase 2 trial

**DOI:** 10.1186/s12916-018-1233-1

**Published:** 2019-01-09

**Authors:** Joaquín Gavilá, Mafalda Oliveira, Tomás Pascual, Jose Perez-Garcia, Xavier Gonzàlez, Jordi Canes, Laia Paré, Isabel Calvo, Eva Ciruelos, Montserrat Muñoz, Juan A. Virizuela, Isabel Ruiz, Raquel Andrés, Antonia Perelló, Jerónimo Martínez, Serafín Morales, Mercedes Marín-Aguilera, Débora Martínez, Juan C. Quero, Antonio Llombart-Cussac, Aleix Prat

**Affiliations:** 1 Fundación Instituto Valenciano de Oncología, Valencia, Spain; 2 Vall d’ Hebron University Hospital/Vall d’Hebron Institute of Oncology, Barcelona, Spain; 3 August Pi i Sunyer Biomedical Research Institute (IDIBAPS), Barcelona, Spain; 4 Hospital Clínic, Barcelona, Spain; 5 SOLTI Breast Cancer Research Group, Barcelona, Spain; 6 Instituto Oncológico Baselga, Hospital Quirón, Barcelona, Spain; 7 Institut Oncològic Rosell, Hospital General Catalunya, Barcelona, Spain; 8 Centro Integral Oncológico Clara Campal, Madrid, Spain; 9 Hospital 12 de Octubre, Madrid, Spain; 10 Hospital Universitario Virgen Macarena, Sevilla, Spain; 11 Hospital Universitario Sant Joan De Reus, Reus, Spain; 12 Hospital Clínico Universitario Lozano Blesa, Zaragoza, Spain; 13 Hospital Universitario Son Espases, Palma, Spain; 14 Hospital Universitario Virgen Arrixaca, Murcia, Spain; 15 Hospital Universitari Arnau Vilanova, Lleida, Spain; 16 Hospital Sagrado Corazón, Barcelona, Spain; 17 Hospital Arnau de Vilanova, Valencia, Spain

**Keywords:** Breast cancer, PAM50, HER2, intrinsic subtypes, cardiac safety, HER2-enriched, neoadjuvant

## Abstract

**Background:**

The Opti-HER HEART trial aimed to optimize activity while minimizing cardiac risk by combining trastuzumab, pertuzumab, and paclitaxel with non-pegylated liposomal doxorubicin in the treatment of HER2-positive early breast cancer.

**Methods:**

Patients with stage II–IIIB HER2-positive breast cancer received neoadjuvant trastuzumab, pertuzumab, paclitaxel, and a non-pegylated liposomal doxorubicin every three weeks for six cycles. The primary endpoint was cardiac safety during neoadjuvant therapy. Type A (symptomatic congestive heart failure) and B (asymptomatic reduction of left ventricular ejection fraction) cardiac events were evaluated. Secondary endpoints included the evaluation of the pathological complete response (pCR) rate and overall response rate, among others. As an ad-hoc exploratory analysis, the expression of 55 breast cancer-related genes, including the *PAM50* genes, was measured in 58 baseline tumor samples and 60 surgical specimens.

**Results:**

Eighty-three patients were recruited. The incidence of cardiac events during neoadjuvant treatment was 2.4%. No type A cardiac event was observed. The overall pCR rate was 56.6% (95% confidence interval (CI) 45.3–67.5%). The HER2-enriched subtype, which represented 52.0% of all baseline samples, was associated with a higher pCR rate compared to non-HER2-enriched tumors (83.3% vs. 46.3%; odds ratio 5.76, 95% CI 1.71–19.42). The association of subtype with pCR was independent of known clinicopathological variables, including hormone receptor status. Compared to baseline samples, surgical specimens showed a significant downregulation of proliferation-related genes (*MKI67* and *CCNB1*) and *ERBB2* levels, and a significant upregulation of luminal-related (*ESR1* and *PGR*) and immune (*CD8A*) genes.

**Conclusions:**

The combination of dual HER2 blockade with trastuzumab and pertuzumab with paclitaxel and non-pegylated liposomal doxorubicin is associated with a low rate of cardiac events. The HER2-enriched subtype is associated with a high rate of pCR.

**Trial registration:**

clinicaltrials.gov, NCT01669239, Registered 20 August 2012.

## Background

Trastuzumab in combination with pertuzumab and chemotherapy improves survival in patients with HER2-positive metastatic breast cancer (BC) [1, 2]. In locally advanced HER2-positive BC, the addition of pertuzumab to trastuzumab-based neoadjuvant chemotherapy (NAC) increases pathological complete response (pCR) rates [3–5]. Moreover, dual HER2 blockage in combination with chemotherapy has been recently explored in the adjuvant setting of early-stage BC in the APHINITY trial [6]. The addition of 1 year of adjuvant pertuzumab to standard chemotherapy plus trastuzumab led to a 19% relative risk reduction in invasive disease-free survival [6]. Based on this data, dual HER2 blockade in combination with chemotherapy is the standard of care in first-line advanced disease and will become more widely used in early BC.

In this context, cardiac dysfunction induced by anti-HER2-based chemotherapy needs attention [7]. In the pivotal first-line metastatic trial that led to the approval of trastuzumab, a New York Heart Association class III–IV cardiac toxicity of 13% and 27% was observed with trastuzumab in combination with paclitaxel or doxorubicin/cyclophosphamide, respectively [8]. According to this data, concomitant administration of trastuzumab and anthracyclines is not recommended. However, recent data suggests that the combination of trastuzumab with anthracyclines might be safe. The use of liposomal anthracyclines, such as pegylated [9] or non-pegylated liposomal doxorubicin (NPLD) [10], have shown equal efficacy in phase III trials compared to conventional anthracyclines in metastatic BC patients. Their formulation offers the advantages of lower specific toxicities and, possibly, an increase in efficacy [11–16]. The data regarding cardiotoxicity were particularly encouraging, with a lower incidence of asymptomatic and symptomatic cardiac toxicity. In 2010, a systematic review on the cardiotoxicity of different anthracycline compounds confirmed that liposomal anthracyclines reduced the overall risk of cardiotoxicity (relative risk 0.38; *p* < 0.001) and the risk of clinical heart failure (relative risk 0.20; *p* = 0.02) [17]. Furthermore, concomitant administration of neoadjuvant trastuzumab with a conventional anthracycline [18] or trastuzumab/pertuzumab with an anthracycline/taxane combination [4] in early BC was found to be associated with a reduction in left ventricular ejection fraction (LVEF) in 4.6% and 5.6% of patients, respectively.

Thus far, no studies have assessed the combination of trastuzumab and pertuzumab with liposomal anthracyclines, and therefore further elucidation of the cardiac safety of dual HER2 blockade in combination with liposomal anthracycline/taxane-based chemotherapy is required. In the Opti-HER HEART trial, we evaluated the cardiac safety and activity following six cycles of trastuzumab and pertuzumab, paclitaxel, and NPLD.

## Methods

### Study design and participants

The Opti-HER HEART trial is an open-label, multicenter, non-randomized, single-arm phase 2 study. Female patients aged between 18 and 74 years with histologically confirmed stage II–IIIB HER2-positive BC eligible for definitive surgery and adequate organic and cardiac function (LVEF ≥ 55%) were enrolled. HER2-positive was defined by the ASCO/CAP 2013 Guidelines and confirmed according to local assessment [19]. Detailed inclusion and exclusion criteria are provided in Additional file 1.

Due to the lack of phase 1 data on the proposed regimen, the trial included a safety run-in consisting of a stage of intensified cardiac and hematological safety monitoring for the first 10 patients [20]. This safety monitoring involved clinical evaluation, an electrocardiogram, weekly hematological evaluation, and LVEF assessment before each cycle of study medication and every three months during the adjuvant period, until completion of 12 months from the day of the first administered study drug. LVEF assessment was determined by echocardiogram or multiple-gated acquisition scan, using the same technique in each patient across all the trial assessments. The pre-planned safety interim analysis was evaluated by an independent data monitoring committee (sponsor representatives, investigators, plus an independent cardiology expert), which deemed the trial safe and supported its continuation to full recruitment. However, primary G-CSF prophylaxis was recommended for the following 73 patients included in the trial.

All patients provided written informed consent, and the protocol was approved by the Ethics Committees from all participating institutions and Spanish Health Authorities. The study was conducted in accordance with Good Clinical Practice principles, the Declaration of Helsinki, and all local regulations.

### Procedures

Patients received six 21-day cycles of NPLD (50 mg/m^2^ intravenously (i.v.) on day 1), paclitaxel (80 mg/m^2^ i.v. on days 1, 8, and 15), trastuzumab (4 mg/kg i.v. cycle 1 on day 1, followed by 2 mg/kg i.v. weekly), and pertuzumab (840 mg i.v. fixed-dose cycle 1 on day 1, followed by 420 mg fixed-dose cycle 2 on day 1 to cycle 6 on day 1) as NAC followed by surgery. Patients who completed study treatment or experienced intolerable toxicity underwent surgery according to local practices. Asymptomatic LVEF reductions were managed according to a protocol-specified cardiac toxicity algorithm (Additional file 2: Figure S1). After surgery, adjuvant treatment was administered as per investigator preference, with a recommendation to complete a total of 1 year of trastuzumab treatment.

### Outcomes

The primary objective was cardiac safety of neoadjuvant treatment assessed by incidence of New York Heart Association class III and IV heart failure (type A cardiac events) [21] and LVEF reduction [21] (10 percentage-points from baseline and to a value of < 50% or any absolute decrease in LVEF ≥ 20%) by echocardiography or multiple-gated acquisition scan (type B cardiac events). LVEF assessments were conducted at screening/baseline, within 2 days before day 1 of cycles 3 and 5 during the neoadjuvant period, within 9 days before surgery, and then every 3 months during the adjuvant treatment period, for a total of 12 months.

Secondary objectives included the assessment of activity, and the overall cardiac and non-cardiac safety and tolerability profile of the regimen up to 1 year after inclusion in the study. Safety was assessed according to Common Terminology Criteria for Adverse Events version 4.0. Activity was based on pCR rate in the breast and lymph nodes (ypT0/is ypN0), overall response rate by imaging per RECISTv1.1 criteria, and breast conservation rate.

### Gene expression

A section of the formalin-fixed paraffin-embedded breast tissue was examined with hematoxylin and eosin staining to confirm the presence of invasive tumor cells and to determine the minimum tumor surface area. Surgical samples without invasive tumor cells were also profiled. At least two 10 μm formalin-fixed paraffin-embedded slides were used to purify total RNA using the High Pure FFPET RNA isolation kit (Roche, Indianapolis, IN, USA). Macrodissection was performed in baseline and surgical samples (when needed) to avoid contamination with normal breast tissue [22]. A minimum of ~100 ng of total RNA was used to measure the expression of 55 BC-related genes using the nCounter platform (Nanostring Technologies, Seattle, WA, USA), including the 50 genes of the PAM50 subtype predictor, androgen receptor, and four immune genes (*CD4*, *CD8*, *PD1* and *PDL1*). Data was normalized using five housekeeping genes, and log2 transformed. Intrinsic molecular subtypes were identified using the research-based PAM50 predictor as previously described [23].

### Statistical analysis

Eighty-three patients were required to reject, with 80% confidence, the null hypothesis that the addition of NPLD to a neoadjuvant regimen containing paclitaxel, trastuzumab, and pertuzumab does not increase the incidence of cardiac events above the historical control [10, 24–28] of 18% (consisting of 3% symptomatic and 15% asymptomatic events), at the 0.05 level of significance. The intention-to-treat population included all enrolled patients. The safety population is the subset of patients who received at least one dose of study treatment and have at least one safety assessment. Analysis of the primary safety outcome included the safety and intention-to-treat population.

The association between two variables was evaluated using Student’s *t* test, Pearson’s χ^2^ test, or Fisher’s exact test. Odds ratio (OR) with a 95% confidence interval (CI) were estimated using univariate and multivariable logistic regression analyses. All statistical tests were two-sided and considered significant when *p* ≤ 0.05. To identify genes differentially expressed between paired baseline and surgical samples, a paired two-class significance analysis of microarrays was used with a false discovery rate (FDR) ≤ 1%. All statistical analyses were performed using the R v3.2.3 software.

## Results

### Clinicopathological characteristics

**Fig. 1:**
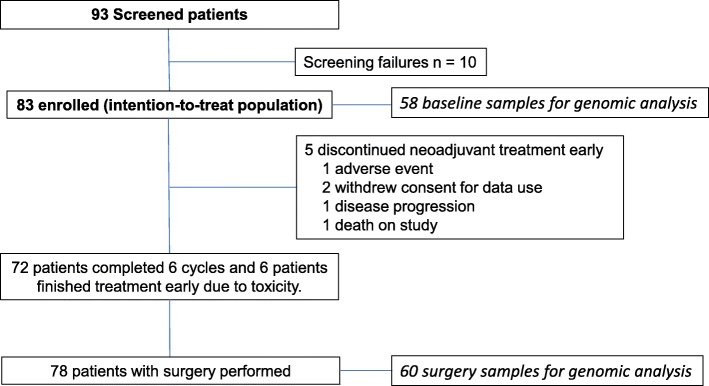
CONSORT diagram

From June 2013 to January 2015, 83 patients with stage II–IIIB HER2-positive BC and adequate cardiac function were enrolled in 18 sites in Spain (Fig. 1). Patient characteristics are summarized in Table 1. The mean age of patients was 49.5 (standard deviation, 10.9) years, with the most frequent age group (*n* = 45; 54.2%) being 45–64 years of age. The mean body mass index was 26.4 kg/m^2^ (standard deviation, 5.9) and 21.7% were active smokers; 9.6% (*n* = 8) of patients were under treatment for arterial hypertension, 4.8% (*n* = 4) for diabetes mellitus, and 9.6% (*n* = 8) for dyslipidemia. Most patients were pre-menopausal (*n* = 54; 65.1%) and had hormone receptor (HR)-positive disease (*n* = 57; 68.7%), clinically node-positive disease (*n* = 39; 46.9%), or T2 tumors (*n* = 70; 84.3%); stage III disease occurred in 21.7% (*n* = 18) of patients.

**Table 1:** Patient demographics at baseline

	*n*	%
	83	–
Age, median and range	49 (22–78)	
Tumor size, median (mm) and range	30 (20–80)	
Clinical nodal status		
N0	40	48.2
N1	30	36.1
N2	8	9.6
N3	1	1.2
Nx	4	4.9
Hormone receptor status		
Negative	26	31.3
Positive	57	68.7
Menopausal status		
Pre-menopausal	54	65.1
Post-menopausal	29	34.9
Tumor stage		
II	65	78.3
III	18	21.7
Histologic grade		
1	5	6.0
2	28	33.7
3	32	38.6
Unknown	18	21.7

### Treatment safety

Overall, 78 (93.9%) patients underwent surgery (Fig. 1), of whom 72 (86.7%) completed six cycles of treatment, and six patients stopped treatment before completion of the six cycles due to toxicity. Overall, five patients discontinued treatment, one (1.2%) due to objective disease progression, one (1.2%) following the first cycle due to a paclitaxel-associated hypersensitivity reaction, and one (1.2%) died after the first cycle due to acute respiratory failure at home; no necropsy was performed*.*

The mean LVEF at baseline was 66% (range 57–88%). For 27.7% (*n* = 23) of patients at least one LVEF assessment was missing or not performed within the protocol-specified interval. In 79.5% (*n* = 67) of patients, the final treatment value (the last available LVEF value up to the end of the study treatment period) was almost unchanged compared with baseline (no change, or increase or decrease from baseline by ten percentage points).

**Fig. 2:**
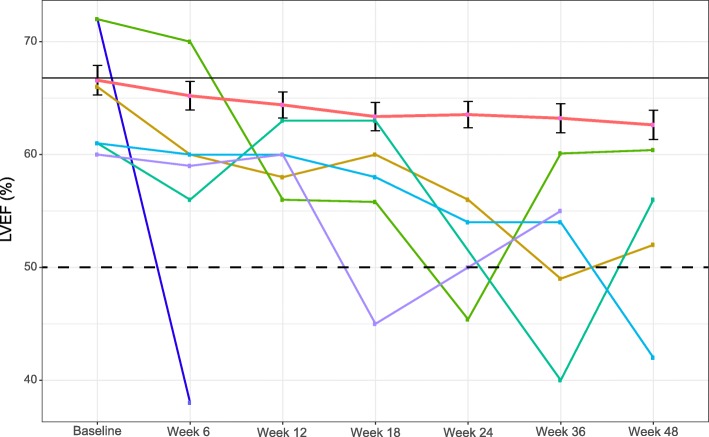
Left ventricular ejection fraction (LVEF) changes during neoadjuvant treatment and adjuvant period: from global population in red and from each of six patients who had > 10% drop in LVEF during the study period

The study met its primary endpoint with an incidence of cardiac events during NAC of 2.4% (95% CI 0.2–8.4%). All cardiac events were type B and occurred in two patients, with an absolute decrease in LVEF up to 45% and 38% (Fig. 2); 1 patient with a type B cardiac event was able to receive standard adjuvant trastuzumab after surgery and the second was lost to follow-up after consent withdrawal. During adjuvant treatment with trastuzumab, 4 further patients (5.1%, 95% CI 1.4–12.6) presented a type B cardiac event; the LVEF value recovered to ≥ 50% in 100% of those patients.

The most frequent grade 3–4 adverse event during NAC was neutropenia (*n* = 28, 34%), which was less frequent in patients receiving G-CSF prophylaxis (25% vs. 67%) and was complicated with febrile neutropenia in five patients (6.0%). Other common adverse events were asthenia (*n* = 11; 13.3%), mucositis (*n* = 8; 9.6%), and diarrhea (*n* = 6; 7.2%). The most common of adverse events are presented in Table 2.

**Table 2:** Most common adverse events

	Grade 1–2		Grade 3		Grade 4	
	*n*	%	*n*	%	*n*	%
Hematologic						
Neutropenia	10	12.0	19	22.9	9	10.8
Anemia	24	28.9	4	4.8	0	0.0
Thrombocytopenia	1	1.2	0	0.0	0	0.0
Febrile neutropenia	0	0.0	4	4.8	1	1.2
Non-hematologic						
Increased ALT concentration	1	1.2	0	0.0	0	0.0
Increased AST concentration	1	1.2	0	0.0	0	0.0
Asthenia/Fatigue	61	73.5	11	13.3	0	0.0
Nausea	46	55.4	4	4.8	0	0.0
Diarrhea	63	75.9	6	7.2	0	0.0
Vomiting	28	33.8	1	1.2	0	0.0
Mucositis	44	53.0	8	9.6	0	0.0
Peripheral edema	2	2.4	0	0.0	0	0.0
Peripheral sensory neuropathy	15	18.1	5	6.0	0	0.0
Dry skin	11	13.2	0	0.0	0	0.0
Left ventricular dysfunction	2	2.4	1	1.2	0	0.0

### Treatment activity

Forty-seven (56.6%; 95% CI 45.3–67.5%) patients achieved a pCR in the breast and axilla. Patients with HR-negative disease had a significantly higher overall pCR rate than patients with HR-positive disease (76.9% vs. 47.3%, OR 3.1, 95% CI 1.2–8.7). No other clinicopathological variable (i.e., age, tumor size, menopausal status, nodal status, or histological grade) was associated with pCR. The overall response rate was 91.3% (*n* = 74 of 81 evaluable patients). Only one patient had disease progression on study treatment and underwent a different therapy. Mastectomy was planned in 35 patients before NAC; of these, 4 (11.4%) patients were able to receive breast conserving surgery after NAC.

### Intrinsic subtype identification at baseline

**Fig. 3:**
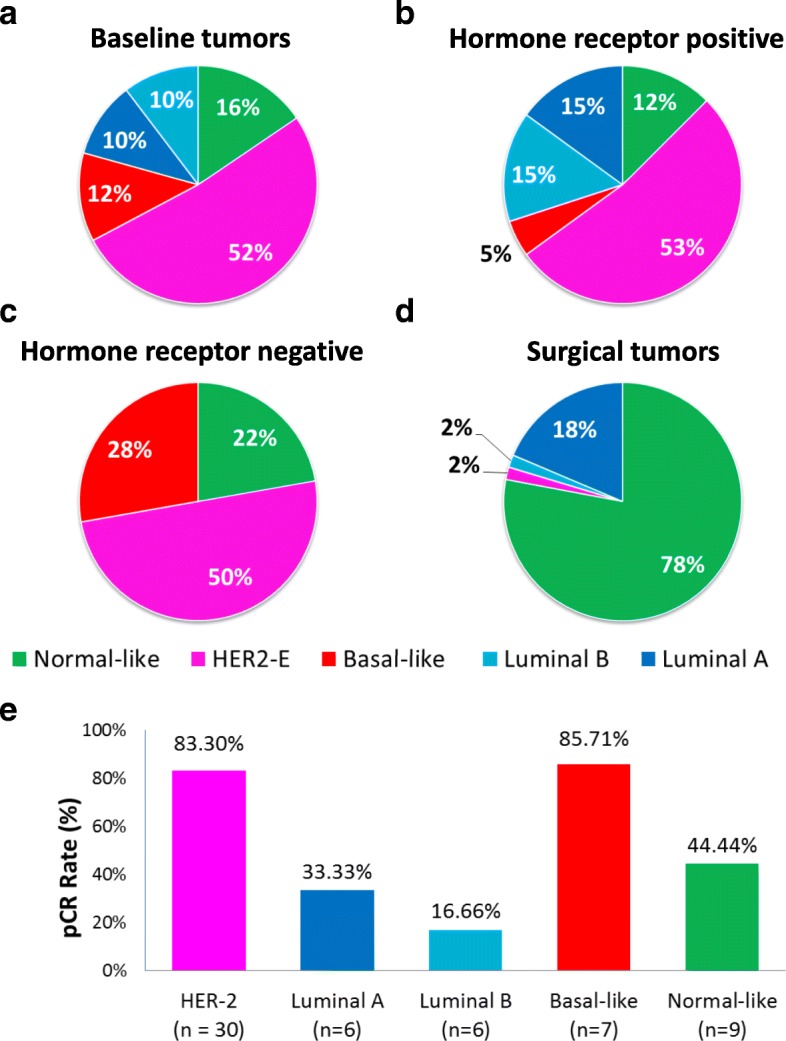
Molecular heterogeneity of HER2-positive breast cancer. Intrinsic subtype distribution in baseline tumors (**a**), according to hormonal receptor status (**b**, **c**), and in surgical tumors (**d**). **e** Pathological complete response (pCR) rates of the intrinsic subtypes identified at baseline

A total of 58 (69.8%) baseline tumors were profiled for gene expression. No statistically significant differences were observed between baseline characteristics of this subset of samples and the overall study cohort (Additional file 3: Table S1). The majority of tumor samples (*n* = 30; 52.0%) were identified as HER2-enriched (Fig. 3a), followed by basal-like (*n* = 7; 12.07%), luminal A (*n* = 6; 10.3%), and luminal B (*n* = 6; 10.3%). As expected, the intrinsic subtype differed between HR-positive (Fig. 3b) and HR-negative (Fig. 3c) disease (*p* = 0.021). Of note, no luminal tumor was identified in HR-negative disease, and only 1 basal-like sample was identified in HR-positive disease. Interestingly, the HER2-enriched subtype was identified in 52.5% (*n* = 21) and 50% (*n* = 9) of HR-positive and HR-negative disease, respectively.

### Intrinsic subtype identification at surgery

A total of 60 (76.9%) surgical specimens were profiled regardless of the type of pathological response (Fig. 3d). The vast majority of samples (*n* = 46; 76.6%) were classified as normal-like, followed by luminal A (*n* = 12; 20%), luminal B (*n* = 1; 1.7%), and HER2-enriched (*n* = 1; 1.7%). As expected, the normal-like subtype was identified in a higher proportion of samples achieving a pCR (89.3%) compared to samples with residual disease (30.7%; *p* < 0.001) (Additional file 4: Figure S2). Five cases (12.2%) that achieved a pCR were identified as luminal A (*n* = 4) and HER2-enriched (*n* = 1).

### Treatment activity based on baseline intrinsic subtype

Rates of pCR varied significantly according to intrinsic subtype (*p* < 0.001) determined at baseline. The highest rate of pCR was observed in the basal-like subtype (85.7%), followed by HER2-enriched (83.3%), normal-like (44.4%), luminal A (33.3%), and luminal B (16.6%). HER2-enriched tumors were found to be associated with higher pCR rates compared with non-HER2-enriched tumors (83.3% vs. 46.5%; OR 5.7, 95% CI 1.7–19.4, *p* = 0.004), even after adjusting for HR status, tumor size, age, and nodal status (OR 13.5, 95% CI 2.5–72.5, *p* = 0.002). Within HR-positive disease, the pCR rates of HER2-enriched and non-HER2-enriched tumors were 76.1% and 31.5%, respectively.

### Treatment activity based on baseline single gene expression

We evaluated the association between the expression (as a continuous variable) of each gene measured at baseline and pCR (Additional file 5: Figure S3). The expression of 8 and 6 genes was found significantly associated with pCR and residual disease, respectively (FDR ≤ 1%). Among them, amplicon 17q12-21 genes, such as *ERBB2* and *GRB7*, and immune genes related to CD8 T-cell infiltration, such as *CD8A* and *PD1*, were associated with a higher likelihood of achieving a pCR. On the contrary, luminal-related genes, such as *ESR1*, *PGR*, *NAT1*, and *BCL2*, were associated with residual disease at surgery.

Additionally, we assessed the association of each individual gene with pCR after adjusting for clinicopathological parameters such as HR status, tumor size, age, and nodal status. Eight of the 14 genes (57.1%) remained significantly associated with pCR (Additional file 5: Figure S3). A similar analysis was performed after adjusting for the previous clinicopathological variables and intrinsic subtype (HER2-enriched vs. non-HER2-enriched). Only *CD8A* remained significantly associated with pCR.

### Single gene expression changes between baseline and surgery

**Fig. 4:**
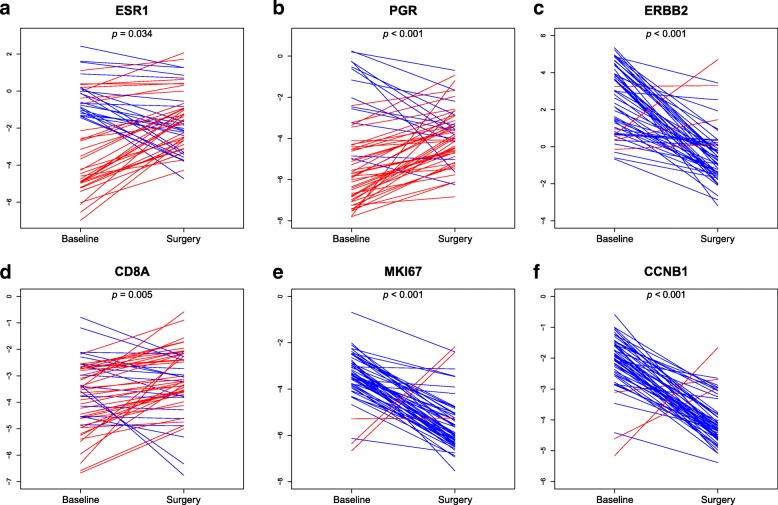
Six differentially expressed genes between baseline and surgical specimens. **a** ESR1, **b** PGR, **c** ERBB2, **d** CD8A, **e** MKI67, **f** CCNB1

To identify genes whose expression changed between baseline and surgical specimens, we performed a paired two-class significance analysis of microarrays. A total of 49 of the 55 genes (89.1%) were found to change their expression between the two time-points (FDR ≤ 1%; Additional file 6: Table S2). Compared to baseline samples, 36 and 13 genes were found to be under- and overexpressed in surgical specimens, respectively. For example, there was an increase in the expression of both hormone receptors (i.e., *ESR1* and *PGR*) and *CD8A*, and a decrease in the expression of *ERBB2* and proliferation-related genes (Fig. 4).

Finally, we repeated the same analysis focusing on 19 surgical samples with residual disease (i.e., after excluding the samples that achieved a pCR). A total of 22 genes were found to have a significant change in expression. Compared to baseline samples, 2 genes (i.e., *EGFR* and *SFRP*1) were more expressed in residual tumors, and 20 genes were less expressed, including *ERBB2* and *MKI67*.

## Discussion

Results from the Opti-HER HEART trial suggest that a regimen that includes the most active drugs for HER2-positive disease (trastuzumab, pertuzumab, paclitaxel, and anthracycline) is effective and is associated with a low rate of cardiac events. Among 83 patients recruited, no episode of symptomatic heart failure was observed, and only 2.4% experienced an asymptomatic cardiac event during NAC. Our results confirm that a dual blockade in combination with anthracycline and taxanes is associated with a low incidence of asymptomatic LVEF decline or symptomatic heart failure [29].

Recently, four further studies have assessed the concurrent administration of trastuzumab and pertuzumab with anthracyclines. In arm A of the phase 2 TRYPHAENA trial, 75 patients received six cycles of trastuzumab and pertuzumab in combination with 5-fluorouracil, epirubicin, and cyclophosphamide (FEC) followed by docetaxel [4]. Four (5.6%) patients experienced a reduction in LVEF of ≥ 10 percentage points from baseline to < 50%, and no patient experienced symptomatic cardiac toxicity. During post-treatment follow-up, 2 out of 72 (2.8%) patients had left ventricular systolic dysfunction of any grade; 8 (11.1%) patients experienced a LVEF reduction of 10% from baseline to < 50% [30]. In the HER2-positive cohort from the GeparSepto trial [31], 396 patients received four cycles of weekly paclitaxel (either solvent-based or nab-paclitaxel, according to randomization) followed by four cycles of epirubicin plus cyclophosphamide, with concurrent trastuzumab and pertuzumab. A LVEF reduction from baseline occurred in 7.6% of patients, with 2.0% of patients showing a reduction to < 50%, representing a ≥ 10% reduction from baseline. Foldi et al. [32] treated 50 patients with weekly paclitaxel followed by FEC in combination with trastuzumab and pertuzumab. Overall, 14 of 50 patients experienced a ≥ 10% reduction in LVEF during the treatment and 48-week cardiac monitoring period. Only one patient had an LVEF reduction to below the limit of 50% [33]. In arm B of the TRAIN-2 study [32], 219 patients were randomized to receive three cycles of FEC followed by six cycles paclitaxel and carboplatin in combination with trastuzumab and pertuzumab concurrent with all chemotherapy cycles. The overall incidence of clinically significant decline in LVEF (10 percentage points from baseline to an absolute value below 50%) was 5%.

Regarding non-cardiac toxicities, the combination was well tolerated; nevertheless, G-CSF administration should be strongly considered due to high rates of grade 3–4 neutropenia without this supportive treatment. Other adverse effects were manageable and are concordant with those reported in the TRYPHAENA [4] and BERENICE trials [34].

The pCR rate of 56.6% obtained in our study is similar to those observed in the TRYPHAENA [4] (56.2% in arm A), BERENICE (61.8% in cohort A) [34], and KRISTINE [35] trials (56.0% in the TCH+P arm). Interestingly, no difference in pCR rates was observed across these studies despite the different proportions of HR-positive versus HR-negative disease. The proportion of HR-positive disease was higher in our study (68.7%) than in the KRISTINE (62%), BERENICE (64.3% in Cohort A), and TRYPHAENA (53.4% in Arm A) trials. The pCR rates according to HR status were also similar across the four studies. In HR-negative disease, the pCR rates were 76.9%, 79.4% (pCR in the breast-only group), 81.5%, and 72.4% in the Opti-HER HEART, TRYPHAENA, BERENICE, and KRISTINE trials, respectively. The pCR rates in HR-positive disease were 47.3%, 46.2% (pCR in the breast-only group), 51.6%, and 44.0% in the Opti-HER HEART, TRYPHAENA, BERENICE, and KRISTINE trials, respectively.

**Fig. 5:**
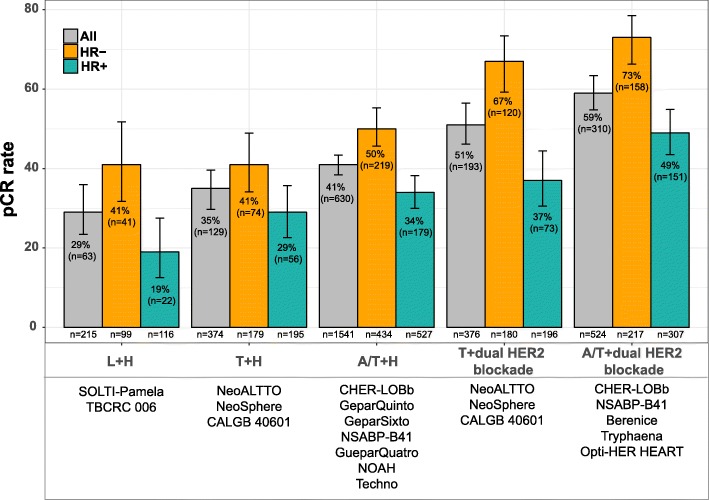
Rates of pathological complete response (pCR) according to the type of chemotherapy and anti-HER2 therapy using data from 15 neoadjuvant clinical trials in HER2-positive breast cancer. Bars denote 95% CIs. *T* taxane, *L* lapatinib, *H* herceptin (trastuzumab), *A/T* anthracycline/taxane based

To contextualize the activity results of the Opti-HER HEART trial, we have reviewed the literature and combined the results of 16 neoadjuvant HER2-positive trials incudling a total of 2923 patients [4, 23, 34, 36–44] (Additional file 6: Table S2). Treatment activity was based on the type of chemotherapy (no chemotherapy, taxane only, or anthracycline/taxane based) and of anti-HER2 treatment (trastuzumab only or dual HER2 blockade). The pCR rates achieved with dual HER2 blockade in the absence of chemotherapy (29.3%; 95% CI 23.4–35.9) in an unselected population were comparable to the pCR rates achieved with a 12-week treatment of a taxane (either docetaxel or paclitaxel) in combination with trastuzumab (34.6%; 95% CI 29.7–39.6) (Fig. 5). The pCR rate achieved with anthracycline/taxane-based chemotherapy in combination with trastuzumab (40.9%; 95% CI 38.4–43.4) was higher than the pCR rate following dual HER2 blockade without chemotherapy (29.3%). Moreover, treatment with a taxane alone in combination with dual HER2 blockade with trastuzumab and lapatinib or pertuzumab obtained higher pCR rates compared to anthracycline/taxane-based chemotherapy in combination with trastuzumab (51.2% vs. 40.9%). Finally, anthracycline/taxane-based chemotherapy in combination with dual HER2 blockade led to higher pCR rates compared to taxane-only in combination with dual HER2 blockade (63.5% vs. 51.2%). These results, together with those from pivotal adjuvant trial [6], suggest that multi-agent chemotherapy and dual HER2 blockade should be considered the standard of care for most patients with early-stage HER2-positive disease, although strategies to de-escalate treatment in HER2-positive disease, such as paclitaxel in combination with trastuzumab, might be considered for most patients with small node-negative disease [45, 46].

Our study further supports that HER2-positive disease is biologically heterogeneous and that this heterogeneity is not completely captured by HR status [23, 47–50]. Among the different intrinsic molecular subtypes of BC, the HER2-enriched was the most prevalent, being significantly associated with higher pCR rates compared to non-HER2-enriched (83.3% vs. 46.4%). Of note, the pCR rate of the HER2-enriched subtype in our study is similar to that obtained with paclitaxel, trastuzumab, and lapatinib in CALGB40601 (83.3% vs. 80.0%) [43], and higher than the pCR rate obtained with lapatinib and trastuzumab without chemotherapy in the SOLTI-PAMELA trial (83.3% vs. 40.6%) [23]. These data suggest that, in the presence of dual HER2 blockade, the HER2-enriched subtype is still chemo-sensitive but might not benefit from additional chemotherapy beyond just a single taxane. Further studies are needed to define the clinical utility of the intrinsic subtypes within HER2-positive disease.

In the last few years, most studies have focused on the intrinsic subtypes of primary untreated HER2-positive tumors [23, 48–51]. However, less is known regarding the subtype distribution in residual tumor samples treated with chemotherapy and anti-HER2 therapy. Similarly to the CALGB40601 study [43], most residual specimens were classified as luminal A or normal-like. Although the normal-like subtype has been associated with lower tumor cellularity at surgery, the presence of luminal A tumors fits with the observed increased expression of *ESR1* and *PGR*, and decreased expression of proliferation-related genes like *CCNB1* and *MKI67*. Whether this is due to changes in the biology of tumor cells at baseline or to selection of clones by NAC cannot be addressed herein and remains unknown. In addition, it is unclear why five cases that achieved a pCR were identified as non-normal subtypes.

Two interesting findings regarding immune gene expression were identified in our study. First, we observed an association between the mRNA expression levels of CD8A and PD1 measured at baseline and the likelihood of achieving a pCR. CD8A provided additional information beyond intrinsic subtype in a multivariable model. This is concordant with findings obtained in tumor samples from the CALGB40601 [43], ACOSOG Z1041 [52], and CHER-LOBb [53] studies using different immune-related gene expression signatures. These data suggest that integration of intrinsic subtype with immune gene expression, or tumor-infiltrating lymphocytes [54, 55], might better predict the probability of achieving a pCR. Second, we observed that immune genes, such as *CD8A* and *PD1*, were significantly upregulated in surgical specimens compared to baseline samples, indicating that immune activation is a dynamic phenomenon during treatment.

A limitation of our trial is that long-term cardiac safety was not evaluated and no survival outcome data is available. Furthermore, given the relatively small sample size, the number of observed events results in a lack of precision of the estimated rate, exacerbated by the fact that patients had a low probability of cardiac events due to their mean age and lack of risk factors.

Moreover, the genomic correlative analyses were exploratory and did not include all specimens of the study (69.9% baseline and 76.9% surgical specimens). Additionally, a rather small number of genes was evaluated. Therefore, we were limited regarding the ability to derive new gene signatures and identify new biological processes associated with treatment response. To overcome this issue, we are currently performing RNA-seq analysis in all samples obtained from this study.

## Conclusions

The combination of trastuzumab, pertuzumab, paclitaxel, and NPLD is associated with a low rate of cardiac events. Among the different molecular subtypes of HER2-positive disease, the HER2-enriched is associated with a very high rate of pCR (~80%). Exploiting the biology of post-treatment samples warrants further investigation.

## Additional files

Additional file 1: Population criteria. Detailed inclusion and exclusion criteria. (PDF 238 kb)

Additional file 2: **Figure S1.** Protocol-specified cardiac toxicity algorithm. (PDF 256 kb)

Additional file 3: **Table S1.** Overall study population and biomarker population characteristics. (PDF 171 kb)

Additional file 4: **Figure S2.** Intrinsic subtypes in surgical specimens. (PDF 130 kb)

Additional file 5: **Figure S3.** Effect of 14 single genes on pathological complete response (pCR) adjusting for clinicopathological parameters. (PDF 184 kb)

Additional file 6: **Table S2.** Rates of pathological complete response (pCR) reported across 15 published neoadjuvant clinical trials in HER2-positive breast cancer. (PDF 283 kb)

## Abbreviations

BC: breast cancer
CI: confidence interval
HR: hormone receptors
i.v.: intravenously
FDR: false discovery rate
FEC: fluorouracil, epirubicin and cyclophosphamide
LVEF: left ventricular ejection fraction
NAC: neoadjuvant chemotherapy
NPLD: non-pegylated liposomal doxorubicin
OR: odds ratio
pCR: pathological complete response
