# Supplementary material for: Safety, activity, and molecular heterogeneity following neoadjuvant non-pegylated liposomal doxorubicin, paclitaxel, trastuzumab, and pertuzumab in HER2-positive breast cancer (Opti-HER HEART): an open-label, single-group, multicenter, phase 2 trial
Source: BMC Med. 2019 Jan 9;17:8. doi: 10.1186/s12916-018-1233-1 (PMC6325829; doi:10.1186/s12916-018-1233-1)
Supplement: Supplementary file 1 — Population criteria. Detailed inclusion and exclusion criteria. (PDF 238 kb) [file 12916_2018_1233_MOESM1_ESM.pdf]

Additional file 1: Population criteria. Detailed inclusion and exclusion criteria

Detailed inclusion and exclusion criteria

Inclusion Criteria

1. Written informed consent for all study procedures according to local regulatory requirements prior to beginning protocol.
2. Female Patients.
3. Age 18-74 years.
4. ECOG Performance Status of 0 or 1.
5. Histologically confirmed, untreated, invasive breast carcinoma eligible for primary definitive surgery.
6. Tumor size >2 cm by clinical or radiological assessment. If it is an inflammatory tumor with a positive biopsy, it is not necessary to measure> 2 cm in a radiological test.
7. HER2-positive invasive BC, defined as: IHC 3+ in >10% immunoreactive cells or HER2 gene amplification by in situ hybridization (FISH,CISH), defined as a ratio of HER2 gene signals to centromere 17 ≥ 2.0.
8. Known hormone receptor status or the possibility of its assessment.
9. Adequate organ function defined as:
    - Absolute Neutrophil Count (ANC) ≥ 1.5 x 10^9/L.
    - Hemoglobin (Hgb) ≥ 9 g/dL.
    - Platelets > 100 x 10^9/L.
    - Creatinine ≤ 1.6mg/dL.
    - ALT and AST ≤ 2.5 x ULN.
    - Alkaline Phosphatase ≤ 5 ULN.
    - Total Bilirubin ≤ 1.5 mg/dL.
10. Baseline LVEF ≥ 55% measured by echocardiogram or MUGA scan.
11. Negative β-HCG pregnancy test (serum) for premenopausal women of reproductive capacity (those who are biologically capable of having children) and for women for less than 12 months after the menopause. All subjects who are biologically capable of having children must agree and commit to the use of a reliable method of birth control from 2 weeks before administration of the first dose of investigational product until 28 days after the last dose of investigational product.
12. Absence of any psychological, familial, sociological or geographical condition potentially hampering compliance with the study protocol and follow-up schedule; those conditions should be discussed with the patient before registration in the trial.

Exclusion criteria

1. Clinical or radiologic evidence of metastatic disease at the time of study entry.
2. Prior chemotherapy, radiotherapy, or surgery for BC, other than excision of tumor in the contralateral breast, and provided that the patient did not previously receive adjuvant radiotherapy or chemotherapy.
3. Subjects with a concurrently active second malignancy, other than adequately treated non-melanoma skin cancers, in situ melanoma or in situ cervical cancer. Subjects with other non-mammary malignancies must have been disease free for at least 5 years.
4. Known or suspected hypersensitivity reaction to any investigational or therapeutic compound or their incorporated substances.
5. Presence of congestive heart failure or LVEF < 55%.
6. Clinically significant (i.e. active) cardiovascular disease, including cerebrovascular accident (< 6 months before enrollment), unstable angina pectoris, myocardial infarction ≤ 6 months before enrollment, uncontrolled hypertension (systolic > 150 mmHg and/or diastolic > 100 mmHg), or high risk uncontrolled arrhythmias.
7. Uncontrolled diabetes mellitus, active peptic ulcer disease or uncontrolled epilepsy.
8. Active uncontrolled infection at the time of enrollment.
9. History of significant comorbidities that, in the judgment of the investigator, may interfere with the conduction of the study, the evaluation of response, or with informed consent.
10. Use of any investigational agent or participation in another therapeutic clinical trial concurrently or in the previous 30 days before the enrollment.
11. Patients who are pregnant or breast-feeding.
12. Women of child bearing potential who are unable or unwilling to use acceptable contraceptive measures.
13. Inability or unwillingness to abide by the study protocol or cooperate fully with the investigator or designee.
