# Supplementary figures and images for: Safety, activity, and molecular heterogeneity following neoadjuvant non-pegylated liposomal doxorubicin, paclitaxel, trastuzumab, and pertuzumab in HER2-positive breast cancer (Opti-HER HEART): an open-label, single-group, multicenter, phase 2 trial
Source: BMC Med. 2019 Jan 9;17:8. doi: 10.1186/s12916-018-1233-1 (PMC6325829; doi:10.1186/s12916-018-1233-1)

Figure S1 - Protocol-specified cardiac toxicity algorithm

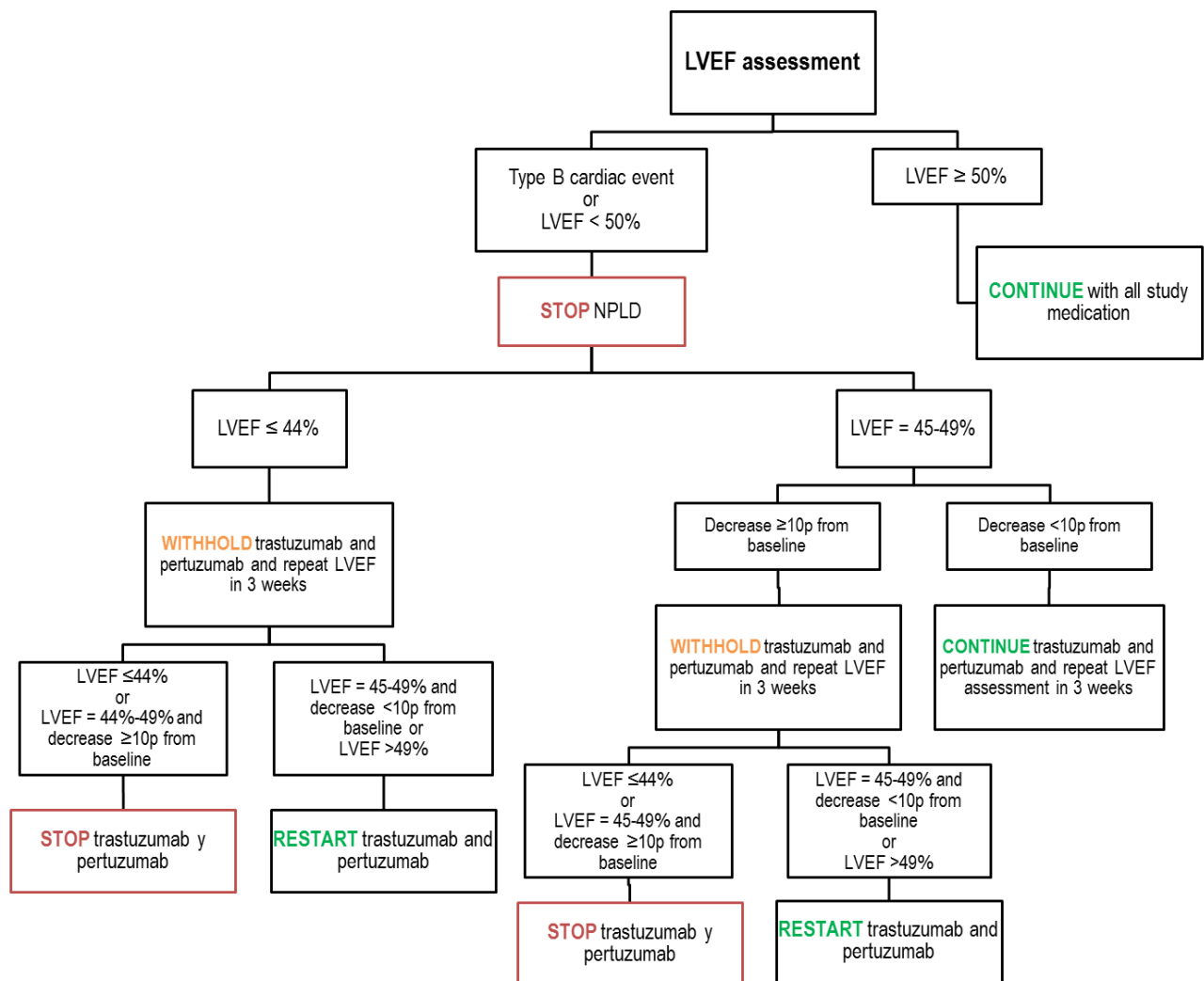

Supplement: Supplementary file 2 — Figure S1. Protocol-specified cardiac toxicity algorithm. (PDF 256 kb) [file 12916_2018_1233_MOESM2_ESM.pdf]
