# Supplementary material for: Safety, activity, and molecular heterogeneity following neoadjuvant non-pegylated liposomal doxorubicin, paclitaxel, trastuzumab, and pertuzumab in HER2-positive breast cancer (Opti-HER HEART): an open-label, single-group, multicenter, phase 2 trial
Source: BMC Med. 2019 Jan 9;17:8. doi: 10.1186/s12916-018-1233-1 (PMC6325829; doi:10.1186/s12916-018-1233-1)
Supplement: Supplementary file 3 — Table S1. Overall study population and biomarker population characteristics. (PDF 171 kb) [file 12916_2018_1233_MOESM3_ESM.pdf]

Table S1 – Overall study population and biomarker population characteristics.

	**All Patients**		**Patient with tumor samples**	
	**N**	**%**	**N**	**%**
	83	-	58	-
**Age, median (range)**	49 (22-78)		49 (22-74)	
**Tumor size (mm), median (range)**	30 (10-80)		30 (10-80)	
**Clinical nodal status**				
N0	40	48.2 %	23	39.6%
N1	30	36.1 %	23	39.6%
N2	8	9.6 %	8	13.8%
N3	1	1.2 %	1	1.7%
Nx	4	4.9 %	3	5.1 %
**Hormone receptor (HR) status**				
Negative	26	31.3 %	18	31.1 %
Positive	57	68.7 %	40	68.9 %
**Menopausal status**				
Pre-menopausal	54	65.1 %	39	67.2 %
Post-menopausal	29	34.9 %	19	32.7 %
**Tumor stage**				
II	65	78.3 %	44	75.9 %
III	18	21.7 %	14	24.1%
**Histologic grade**				
1	5	6.0 %	3	5.1 %
2	28	33.7 %	15	28.8 %
3	32	38.6 %	27	46.5 %
Unknown	18	21.7 %	13	22.4 %
