# Supplementary material for: Safety, activity, and molecular heterogeneity following neoadjuvant non-pegylated liposomal doxorubicin, paclitaxel, trastuzumab, and pertuzumab in HER2-positive breast cancer (Opti-HER HEART): an open-label, single-group, multicenter, phase 2 trial
Source: BMC Med. 2019 Jan 9;17:8. doi: 10.1186/s12916-018-1233-1 (PMC6325829; doi:10.1186/s12916-018-1233-1)
Supplement: Supplementary file 4 — Figure S2. Intrinsic subtypes in surgical specimens. (PDF 130 kb) [file 12916_2018_1233_MOESM4_ESM.pdf]

Figure S2 - Intrinsic subtypes in surgical specimens

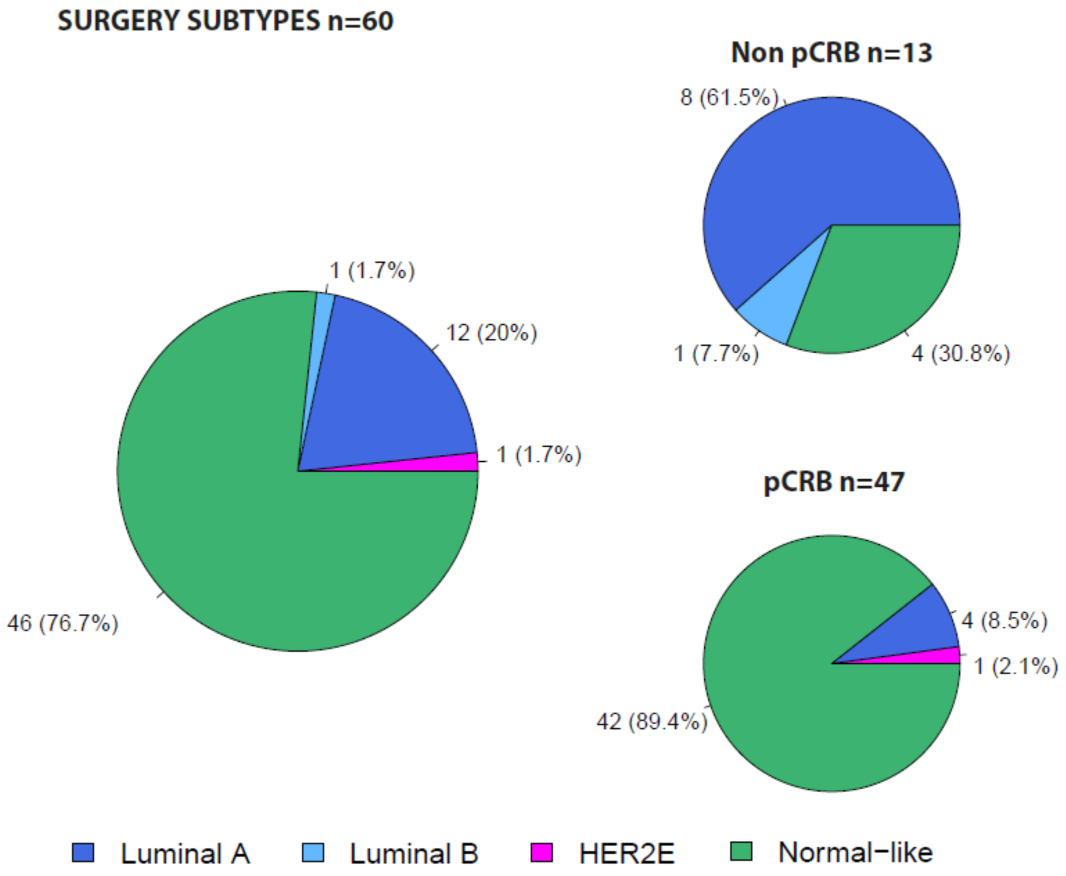

HER2-E: HER2-enriched; LUM A: luminal A; LUM B: luminal B; pCR$_B$: pathologic complete response in the breast.
