# Supplementary material for: Safety, activity, and molecular heterogeneity following neoadjuvant non-pegylated liposomal doxorubicin, paclitaxel, trastuzumab, and pertuzumab in HER2-positive breast cancer (Opti-HER HEART): an open-label, single-group, multicenter, phase 2 trial
Source: BMC Med. 2019 Jan 9;17:8. doi: 10.1186/s12916-018-1233-1 (PMC6325829; doi:10.1186/s12916-018-1233-1)
Supplement: Supplementary file 5 — Figure S3. Effect of 14 single genes on pathological complete response (pCR) adjusting for clinicopathological parameters. (PDF 184 kb) [file 12916_2018_1233_MOESM5_ESM.pdf]

Figure S3 - Effect of 14 single genes on pCR adjusting for clinicopathological parameters.

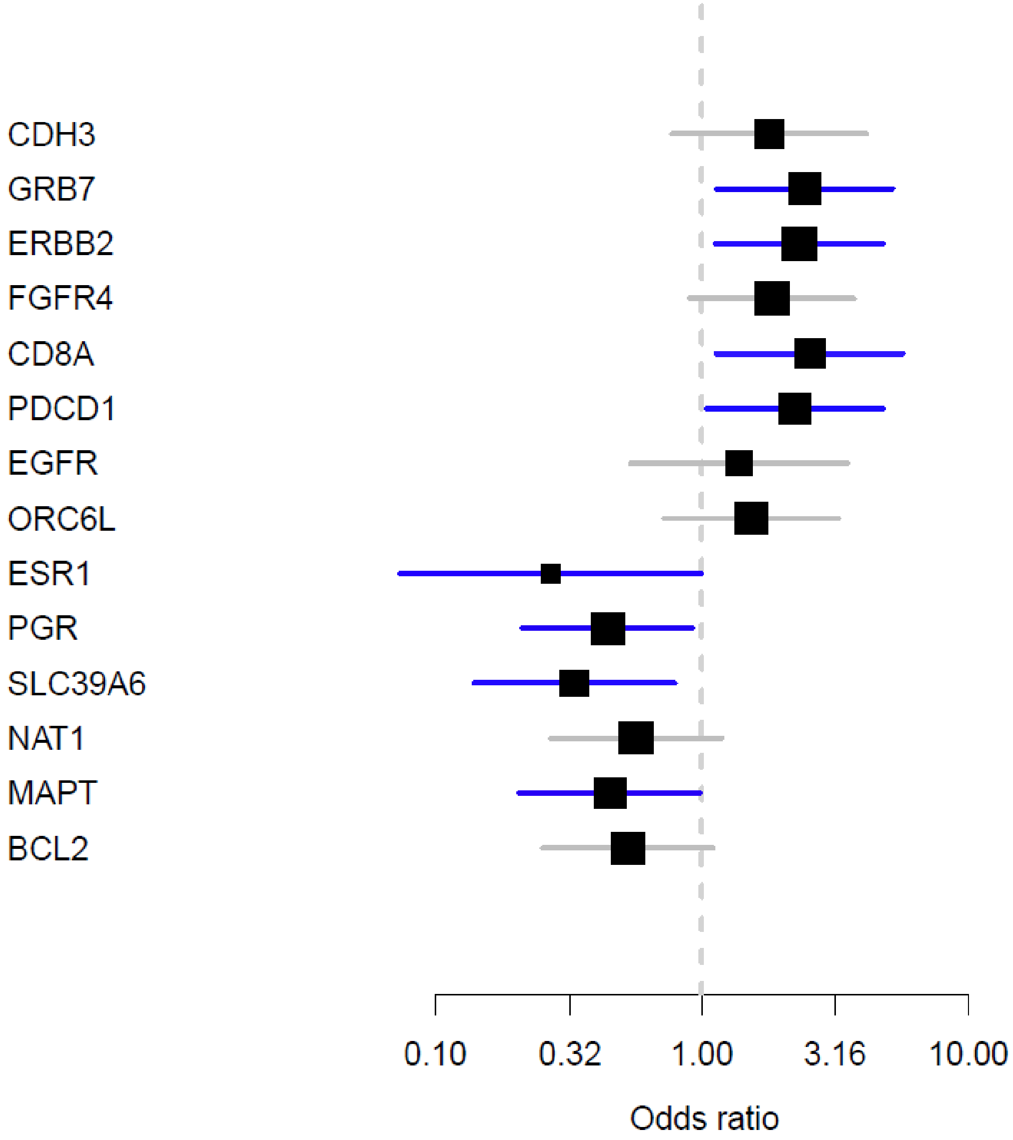

A false discovery rate < 0.01 is considered significant. Each gene has been standardized to have a mean of 0 and a standard deviation of 1. Square indicate odds ratio (OR), and error bars, 95% confidence interval (CI). Square size is inversely proportional to the size of the CI.
