# Supplementary material for: Safety, activity, and molecular heterogeneity following neoadjuvant non-pegylated liposomal doxorubicin, paclitaxel, trastuzumab, and pertuzumab in HER2-positive breast cancer (Opti-HER HEART): an open-label, single-group, multicenter, phase 2 trial
Source: BMC Med. 2019 Jan 9;17:8. doi: 10.1186/s12916-018-1233-1 (PMC6325829; doi:10.1186/s12916-018-1233-1)
Supplement: Supplementary file 6 — Table S2. Rates of pathological complete response (pCR) reported across 15 published neoadjuvant clinical trials in HER2-positive breast cancer. (PDF 283 kb) [file 12916_2018_1233_MOESM6_ESM.pdf]

Table S2 - Rates of pathological complete response (pCR) reported across 15 published neoadjuvant clinical trials in HER2-positive breast cancer.

		All Patients		RH+		RH-negative		
**Study Name**	**Type of Treatment**	**N**	**pCR**	**N**	**pCR**	**N**	**pCR**	**Type of pCR**
CHERLOB	A/T+H	36	25%	21	24%	15	27%	Breast+Axilla
GEPARQUINTO	A/T+H	307	30%	NA	NA	NA	NA	Breast+Axilla
NSABP-B41	A/T+H	176	49%	121	46%	55	58%	Breast+Axilla
GEPARSIXTO	A/T+H	273	51%	NA	NA	NA	NA	Breast+Axilla
GEPARQUATTRO/NOAH/TECHNO	A/T+H	749	40%	385	31%	364	50%	Breast+Axilla
NSABP-B41	A/T+H+L	171	60%	108	55%	63	70%	Breast+Axilla
CHERLOB	A/T+H+L	45	47%	28	36%	17	59%	Breast+Axilla
TRYPHAENA	A/T+H+P	225	62%	114	48%	111	76%	Breast+Axilla
OPTI-HER	A/T+H+P	83	57%	57	47%	26	77%	Breast+Axilla
BERENICE	A/T+H+P	400	62%	252	54%	140	74%	Breast+Axilla
PAMELA	H+L	151	31%	77	18%	74	43%	Breast
TBCRC006	H+L	64	27%	39	21%	25	36%	Breast
NeoALTTO	T+H	149	30%	75	23%	74	37%	Breast
NeoSphere	T+H	107	29%	50	20%	57	37%	Breast
CALGB40601	T+H	118	46%	70	41%	48	54%	Breast
NeoALTTO	T+H+L	152	51%	77	42%	75	61%	Breast
CALGB40601	T+H+L	117	56%	69	41%	48	79%	Breast
NeoSphere	T+H+P	107	46%	50	26%	57	63%	Breast
		**All Patients**		**nonHER2-E**		**HER2-E**		
		**N**	**pCR**	**N**	**pCR**	**N**	**pCR**	**Type of pCR**
PAMELA	H+L	151	30%	50	10%	101	41%	Breast
CALGB40601	T+H/L/LH	265	42%	183	31%	82	66%	Breast+Axilla
NOAH	T+H	63	44%	29	34%	34	53%	Breast+Axilla
NEOALTTO	T+H/L/LH	254	35%	144	22%	110	52%	Breast
OPTI-HER	A/T+H+P	58	57%	28	47%	30	83%	Breast+Axilla
CHERLOB	A/T+H/L/LH	69	28%	47	17%	22	50%	Breast+Axilla
BERENICE	A/T+H+P	400	62%	119	46%	172	74%	Breast+Axilla

T, taxane; L, lapatinib; H, Herceptin (trastuzumab); A/T, anthracycline/taxane-based.
